# Development of a multi-parametric MRI platform to evaluate steady-state water content and optical properties in multiple *ex vivo* bovine lens

**DOI:** 10.1016/j.exer.2025.110822

**Published:** 2025-12-23

**Authors:** Xingzheng Pan, Yadi Chen, Beau Pontre, Catherine Morgan, Jin Jin, Chen Qiu, Courtney A. Thorne, Thomas W. White, Paul J. Donaldson

**Affiliations:** a Department of Physiology, School of Medical Sciences, New Zealand Eye Centre, University of Auckland, New Zealand; b Department of Anatomy and Medical Imaging, School of Medical Science, Faculty of Medical and Health Sciences, University of Auckland, New Zealand; c School of Psychology and Centre for Brain Research, The University of Auckland, Auckland, New Zealand; d Centre for Advanced MRI, Auckland UniServices Limited, Auckland, New Zealand; e Siemens Healthineers Pty Ltd, Australia; f Department of Physiology and Biophysics, Stony Brook University, Stony Brook, NY, USA

**Keywords:** Magnetic resonance imaging, Contrast agent, Lens, Physiological optics, Free water, Bound water, Water content, Multi-parametric mapping

## Abstract

Here we describe the development of magnetic resonance imaging (MRI) protocols which when run on a standard clinical 3T MRI system can non-invasively monitor changes in the water content and refractive properties of bovine lenses maintained under organ culture. Fresh bovine lenses were incubated in artificial aqueous humour and placed in a custom holder within a hand/wrist coil. Multi-parametric mapping sequences (T1, T2*, proton density [PD], and magnetisation transfer [MT] ratio) were optimised to quantify the total, free, and bound water compartments in distinct lens regions (outer cortex, inner cortex, and nucleus). Repeated scanning on different days demonstrated robust inter-sample reproducibility. MRI-derived T2* and MT ratio values were calibrated against refractive index (*n*) profiles calculated using laser ray tracing and subsequently applied to ZEMAX optical modelling to calculate lens power. The imaging protocol achieved high spatial resolution and reproducibility, which allowed the detection of regional variations in water composition within a practical scan time. Hypotonic lens swelling induced significant increases in total and free water across all lens regions but had no effect on bound water or the refractive index gradient. Lens swelling increased lens power (ΔP = 2.98D, p = 0.04) primarily due to a change in the surface geometry of the lens. This ability to quantify lens water states and refractive changes across multiple lens samples will facilitate the screening of novel pharmacological reagents that modulate lens water transport, allowing for testing of their efficacy as potential anti-cataract therapies.

## 1. Introduction

The ability of the ocular lens to correctly focus light onto the retina requires that the lens not only remain transparent but also contribute to the refractive power of the eye. The refractive power of the lens is determined by its surface curvatures (geometry) and the internal gradient of refractive index (GRIN) profile (Donaldson et al., 2017), which is initially established by the overexpression of crystallin proteins in different regions of the lens (Pierscionek, 1989). However, the lens should not be considered as a passive optical element. Instead, evidence indicates that it operates an internal microcirculation system which generates a circulating fluxes of ions and water that not only maintains ionic and fluid homeostasis in the lens, but also deliver nutrients to the nucleus of the avascular lens more rapidly than would be expected though passive diffusion alone (Mathias et al., 2007; Vaghefi and Donaldson, 2018). These circulating fluxes of ions and water have been subsequently shown to be dynamically regulated by a dual feedback system (Gao et al., 2015). This feedback system helps maintain lens homeostasis by ensuring that transient changes in ion and water fluxes do not alter the steady-state refractive power of the lens (Vaghefi et al., 2015; Chen et al., 2022). Taken together, these observations suggest that the steady-state water content of the lens is a crucial parameter that determines the overall functionality of the lens.

The current understanding of water transport in the lens has in part been driven by use of magnetic resonance imaging (MRI), to provide non-invasive measurements of changes in water state initially in *ex vivo* bovine lenses in response to physiological perturbations (Vaghefi, et al., 2011, 2015; Lim et al., 2016) and subsequently *in vivo* experiments performed on both mice (Muir et al., 2020; Pan et al., 2023) and human subjects of different ages (Lie, et al., 2020, 2021). Across these different studies, the MRI parameter longitudinal relaxation time (T1) has been used as a surrogate for free water (Vaghefi et al., 2015; Lie et al., 2021; Pan et al., 2023), while proton density (PD) has been used to indicate the total water content (Dobretsov et al., 2013; Lie, et al., 2021, 2024). The transverse relaxation time (T2) has been used as an index of the bound water-to-protein ratio and, after calibration, converted into refractive index (*n*) values to generate the GRIN profile (Jones et al., 2005; Lie et al., 2020; Muir et al., 2020). The lens geometry and GRIN obtained by MRI can then be used by optical modelling software to assess optical measurements of the lens and how they change in response to experimental perturbations (Vaghefi et al., 2015; Lim et al., 2016; Muir et al., 2020), increasing age (Lie, et al., 2020, 2021), and in mice after genetic manipulation of lens transport proteins (Pan, et al., 2023, 2024). These findings identify lens water content as a key biomarker of age-related optical changes, highlighting the potential of MRI to track changes in free and bound water content that precede the onset of presbyopia and age-related cataract (Donaldson et al., 2017, 2023).

In this study, we have taken the initial steps towards adapting our MRI-based protocols for application to multiple cultured bovine lenses, with the long-term aim of identifying pharmacological agents that modulate lens microcirculation and thereby alter steady-state water content to delay presbyopia and cataract progression in humans. To investigate the effects of modulation of lens microcirculation on the state of lens water, we have introduced a multi-parametric MRI (MPM) imaging protocol incorporating PD (total water) and T1 (free water) mapping adapted from our previous clinical lens studies (Lie et al., 2021, 2024), alongside T2* and the magnetisation transfer (MT) ratio (Tabelow et al., 2019). Rather than conventional T2, which reflects the water-to-protein ratio, our protocol uses T2*, an apparent transverse relaxation time that accounts for field inhomogeneities and is therefore shorter than the conventional T2 (Chavhan et al., 2009). Complementary to this, the MT ratio serves as a semi-quantitative marker of bound water, as demonstrated in other highly ordered tissues, such as myelin (Callaghan et al., 2014; Carey et al., 2018). However, MT related parameters have not previously been applied to the lens, where bound water plays a central role in maintaining the GRIN (Pierscionek and Regini, 2012).

To support this approach, we designed and tested an MRI-compatible multi-lens sample holder, which fits within a standard hand/wrist coil and can be scanned using a 3T clinical MRI. To test the utility and reproducibility of our approach, we exposed the lens to a non-physiological osmotic challenge designed to alter lens water content. Furthermore, by performing parallel laser ray tracing experiments to calculate the GRIN of the bovine lens, we have created a calibration curve that allows T2* and MT values to be converted into the refractive index (*n*), thereby enabling the calculation of the power of the bovine lens. These proof-of-principle experiments demonstrate the utility of our protocols for simultaneously scanning multiple bovine organ cultures under different conditions to detect changes in the spatial distribution of different water states and how these changes affect the overall refractive power of the lens. The multi-lens scanning and analysis platform introduced in this study provides the means to more efficiently screen for therapeutic agents, which, by modulating lens water content, could potentially preserve the optical quality of the ageing human lens.

## 2. Methods

### 2.1. Preparation of bovine lenses

Lenses were dissected from bovine eyes obtained fresh from a local meat processor. Lenses were immediately transferred into the wells of a MRI compatible custom designed sample holder that contained Artificial Aqueous Humour (AAH - NaCl 125 mM; KCl 4.5 mM; MgCl_2_ 0.5 mM; CaCl_2_ 2 mM; NaHCO_3_ 10 mM; glucose 5 mM; Sucrose 20 mM; buffered with 10 mM HEPES to pH 7.1, osmolarity 300 osmol/L). Lenses were placed in pre-warmed AAH supplemented with 1 % antibiotics (penicillin, streptomycin, and neomycin) at 37 °C with 5 % CO_2_ and kept in an incubator for up to 1 h prior to the experiment. To test the intra-day repeatability, two batches of lenses were prepared for each scan (N = 12 for each). Furthermore, to assess the feasibility of the MPM protocols, a hypotonic solution (Hypotonic AAH - NaCl 76.8 mM; KCl 4.5 mM; MgCl2 0.5 mM; CaCl2 2 mM; NaHCO3 10 mM; glucose 5 mM; sucrose 20 mM, 10 mM HEPES, osmolarity 220 osmol/L) was used to induce lens swelling by altering the extracellular osmolality. The osmolality of all solutions was measured by a vapour pressure osmometer (Model 5520, Hyland Scientific, Stanwood, WA, USA).

### 2.2. Sample holder design

**Fig. 1.**
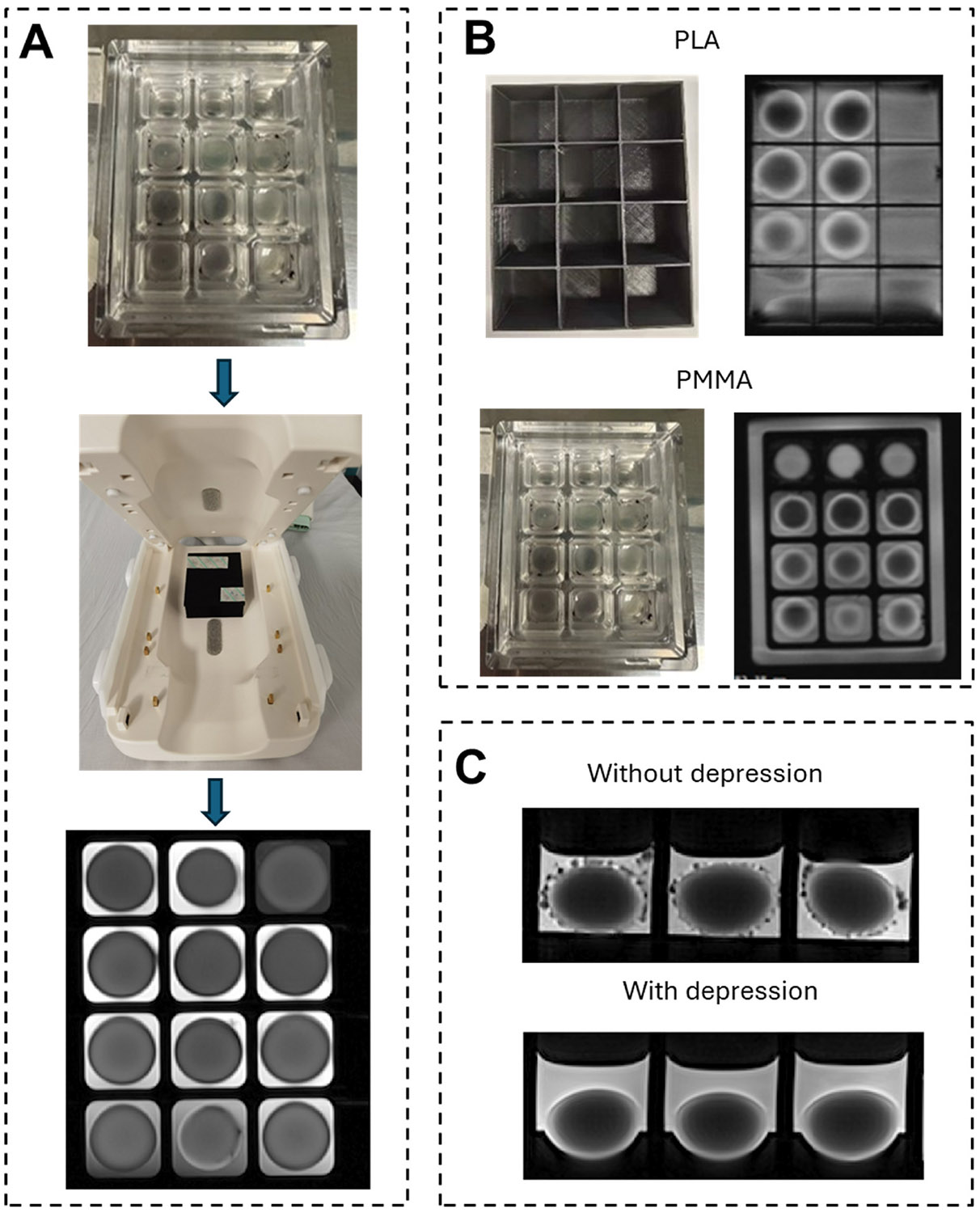
MRI-compatible holder designed to allow imaging of multiple *ex vivo* bovine lenses. **(A)** Custom-designed holder made from transparent PMMA capable of accommodating up to 12 bovine lenses. The holder was sized to fit a 16–channel hand/wrist coil, and transparency aids placement of lenses. **(B)** Images (left) and MRI scans (right) of two sample holders made of PLA (*top*) and the more MRI-compatible PMMA (*bottom*). Under the same multi-parametric MRI protocols, the holder constructed from PMMA produced fewer material-induced interface artefacts than the chamber printed from PLA. **(C)** Axial MRI scans taken through one row of the PMMA holder without (top) and with (bottom) a shallow depression that mirrored the posterior surface of the bovine lens. The holder with the depression facilitated the alignment of the lenses within each row, standardised the positioning of the lens in each well of the chamber, and enabled the reliable acquisition of axial images of the lens.

Lenses were placed in a custom-designed sample holder that accommodated up to 12 lenses in individual wells (Fig. 1A). Several designs were trialled to reduce imaging artefacts, but only the final design is reported here. This sample holder was constructed from the MRI-compatible material polymethyl methacrylate (PMMA), which consisted of 40 % PMMA, 57 % alumina trihydrate (ATH), and 2.7 % stabiliser. A 5 mm deep channel that surrounded the inner region of the chamber was introduced to the design, and when filled with water, minimised susceptibility mismatch artefacts that arose at the interface between the holder and the surrounding air. The PMMA exhibited a light transmittance of 93 %, allowing direct visualisation of lenses when placed in the holder. Each well measured 25 mm in height, 20 mm in width, and 20 mm in length, ensuring that the lenses were fully immersed in bathing media. The bottom of the sample holder was shaped to match the posterior curvature of the lenses, ensuring that the lenses were aligned in the chamber when scanned in the axial plane. The sample holder containing the organ-cultured lenses was then placed in a 16-channel hand/wrist coil (Siemens, Germany), which was positioned in a 3T clinical MRI scanner (MAGNETOM Vida, Siemens Healthineers, Forchheim, Germany) located in the Centre for Advanced MRI (CAMRI) at the University of Auckland.

### 2.3. MPM protocols

The parameters of the MPM protocol, comprising T1 and PD measurements to quantify free and total water, along with T2* and MT measurements to investigate water bound to protein. T1 and PD mapping were performed using a dual-flip angle (FA) protocol with volumetric interpolated breath-hold examination (VIBE) sequences. The selected flip angles (α) were 4° and 23°, consistent with our previously applied clinical protocols (Lie, et al., 2021, 2024). T2* and MT mapping consisted of three multiple-echo FLASH scans with different weightings: T1-weighted (T1W), proton density-weighted (PDW), and magnetisation transfer-weighted (MTW), as described previously (Leutritz et al., 2020). RF receive field ($B_{1}^{-}$) inhomogeneity was corrected automatically using the vendor-supplied normalisation option (“Prescan”). Additional B1 maps were acquired to correct for RF transmit ($B_{1}^{+}$) inhomogeneities. These corrections are essential for accurate parameter quantification (Preibisch and Deichmann, 2009; Chung et al., 2010). The MT preparation pulse in MTW image acquisition was achieved by the default setting in the Siemens’s protocol (Leutritz et al., 2020). Parallel imaging with an accelerator factor of two and partial Fourier acquisition with a factor of 6/8 was applied in these protocols to facilitate rapid image acquisition. General imaging parameters of the MPM protocol are recorded in Table 1.

### 2.4. Data processing

#### T1 & PD calculations:

The low-resolution $B_{1}^{+}$ map was resliced and co-registered with one of the volumetric sets (*a* = 23°). The pixel-wise correction was then performed to correct the signal biased by field inhomogeneity using equation (1) (Venkatesan et al., 1998):

(1) $$\frac{S}{sin(\alpha b_{1})}=\frac{S}{cos(\alpha b_{1})}e^{-\frac{T_{R}}{T_{1}}}+M_{0}\left(1-e^{-\frac{T_{R}}{T_{1}}}\right)$$

Where S is the signal intensity with respect to *a*, *b*_1_ is a multiplier that denotes the ratio of actual α (biased by the field inhomogeneity) and ideal α, which is calculated from the acquired $B_{1}^{+}$ maps (Klose, 1992). After the signal correction, pixel-wise T1 maps were obtained from linear fitting. After the signal correction, T1 and proton density (PD, denoted in *M*_0_ in Eq. (1)) maps were obtained from linear fitting. *PD_water_* was calculated from pure water, which served as a reference for calculating the tissue water content (Lie, et al., 2021, 2024), with the total water content of the tissue, ρ_tissue_ being determined by equation (2):

(2) $$\rho_{\text{tissue}}=\frac{PD_{tissus}}{PD_{water}}$$

#### T2* & MT calculations:

Processing of MPM data was performed using the hMRI-Toolbox (https://github.com/hMRI-group/hMRI-toolbox/wiki), an open-source toolbox developed for quantitative MRI (qMRI) data processing (Tabelow et al., 2019), which is part of the Statistical Parametric Mapping tool (SPM 12, https://www.fil.ion.ucl.ac.uk/spm/software/spm12/) contained in MATLAB (R2023b, The MathWorks, Inc., Natick, MA, USA). In brief, quantitative T2* and MT maps were generated using the hMRI toolbox, employing established methods (Helms et al., 2008). T2* maps were calculated from multiple echoes fitting using all echo images of all T1W, PDW and MTWs (Weiskopf et al., 2014). The MT ratio calculated herein is a semi-quantitative metric that delineates the percentage loss of magnetisation due to the MT-prepared pulse (Helms et al., 2008, 2010). T2* and MT calculations were done with additional $B_{1}^{+}$ corrections for the respective RF transmission inhomogeneity. The detailed mathematical descriptions of T2* and MT ratio calculation can be found in (Tabelow et al., 2019).

#### Configuration of the hMRI toolbox:

The hMRI toolbox used in this study was initially designed for neuroimaging; therefore, several default settings were modified to optimise it for use on the lens. In particular, the thresholds of R2* (1/T2*) and MT ratio in the default toolbox were set to the following values: R2 = 10 s^−1^, MT ratio = 30, to compensate for the very low T2* and MT ratio in the lens caused by the high protein content that exists in the central lens. Detailed steps for toolbox adaptation can be found on the hMRI-toolbox web page (Tabelow et al., 2019).

#### Regional analysis:

**Fig. 2.**
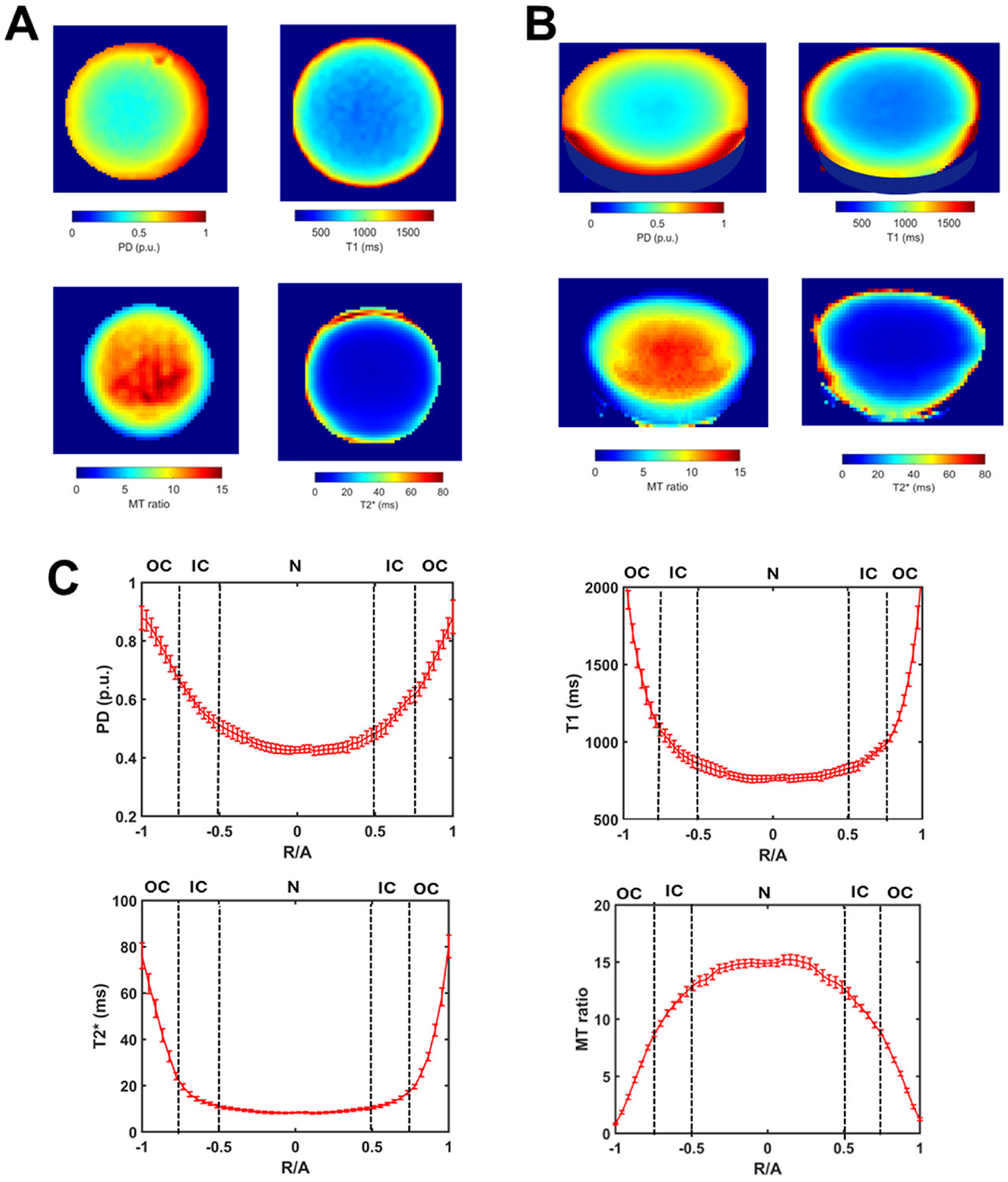
Multiparametric MRI maps extracted from *ex vivo* organ-cultured lens. Images from a representative lens taken through the lens equator **(A)** and poles (**B**) showing colour-coded maps of PD, T1, MT and T2* values. The colour maps show that T1 (free water), T2* (water-bound-protein ratio), and PD (total water) values are highest in the outer cortex of the lens (red) and lowest in the nucleus (blue). In contrast, the MT ratio, reflecting bound water, is lowest in the outer cortex (blue) and highest in the nucleus (red). (C) Line profiles extracted from the equatorial maps shown in **A** for PD, T1, MT and T2* are plotted against normalised lens distance (*r/a*). To facilitate the inter-sample reproducibility analysis (Table 2), profiles were divided into three defined regions: outer cortex, OC (±< *1 r/a* > ±0.75), inner cortex, IC (±> 0.5 *r/a* <±0.75), and nucleus, N (−0.5 < *r/a* < 0.5).

**Fig. 3.**
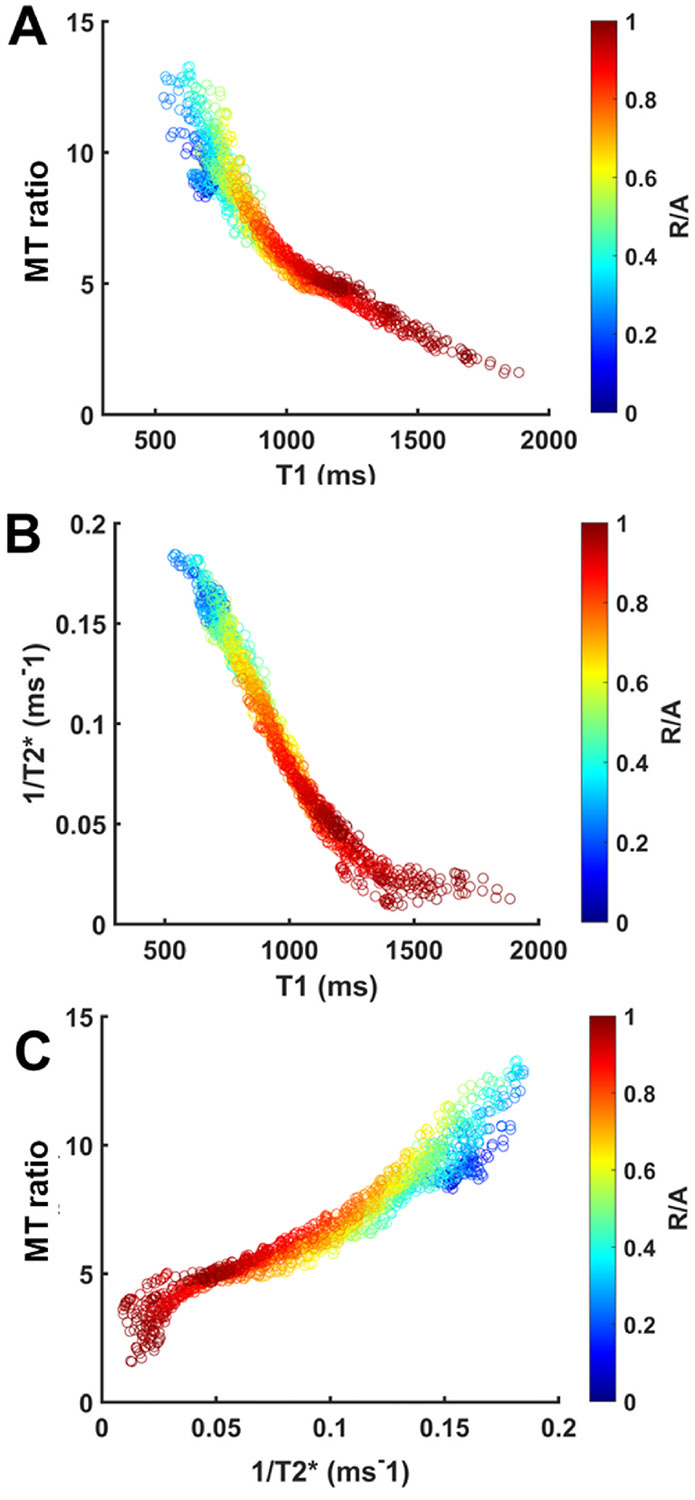
Relationships between different states of water in the bovine lens. (**A & B**) Plots of T1 versus MT (**A**) and 1/T2* (**B**) showing a negative correlation where free water (T1) is high and bound water (MT and 1/T2*) is low, and vice versa. (**C**) Plot of MT versus 1/T2* confirming the positive relationship between these two parameters. Normalised lens distance (*r/a*) has been colour-coded (*blue* – *nucleus, N; outer cortex, OC* – *red*) to represent spatial information.

Values were extracted along the equatorial axis using a three-pixel-wide averaging band and plotted against normalised distance (*r/a*) (Fig. 2C), where 1 and 0 represent the lens periphery and centre, respectively (Vaghefi et al., 2015; Muir et al., 2020; Lie et al., 2021). Error bars represent the standard error of the mean. When comparing groups, data were obtained from three locations along the trend profile, defined as outer cortex, OC (±< *1 r/a* > ±0.75), inner cortex, IC (±> 0.5 *r/a* < ±0.75), and nucleus, N (−0.5 < *r/a* < 0.5), to investigate regional differences (Vaghefi et al., 2015a,b). To enhance the visualisation of regional differences in the measured parameters (Fig. 3), each pixel from the MRI scans was assigned a colour based on its spatial location (*r/a*) within the lens. Red denotes pixel values originating from the peripheral cortex (*r/a* = 1), and the colour gradually transitions towards dark blue at the lens centre (*r/a* = 0). Pixel values were then pooled to generate correlation plots with spatial colour encoding (Fig. 3). The inter-sample reproducibility was assessed by two independent sample t-tests with a significance level of 0.05. The coefficients of variation (CoV) were calculated by dividing the standard deviations by the within-subject means.

### 2.5. Retrieval of the GRIN by laser ray tracing

To correlate the MRI-derived parameters with the lens GRIN profiles, we used a custom-built laser ray tracing (LRT) system to measure the GRIN of the bovine lens (Qiu et al., 2017; Chen et al., 2022). This system consisted of a 514-nm laser diode (5 mW, Coherent StingRay, Palo Alto, CA) mounted on a computer-controlled rotation stage (T-RS60A, Zaber Technologies, Vancouver, BC, Canada), which could deliver rays at different projection angles. The stage was mounted on two linear stages (T-LSM200 and T-LSM025, Zaber Technologies, Vancouver, BC, Canada). The 200-mm stage was used to move the laser across the lens for parallel rays, while the 25-mm stage with a side camera (Canon EOS 1100 D, Tokyo, Japan) was used to ensure alignment. A front-facing camera (Canon EOS 1100 D, Tokyo, Japan) was used to capture ray paths and lens geometry. Lenses were mounted with the anterior side up in a stainless-steel holder within a chamber filled with AAH at 34 °C.

The GRIN was reconstructed by passing 151 parallel rays at four projection angles (60°, 70°, 80°, 90°) with alignment checks at each angle. Calibration with a phantom image converted pixels to millimetres. Lens geometry (anterior/posterior radii, axial thickness, equatorial radius) was extracted by fitting aspheric lens equations to sagittal images. The retrieved lens geometrical parameters together with the ray trajectories were then used as inputs to an iterative computational minimisation algorithm to retrieve the lens GRIN (Qiu et al., 2017).

### 2.6. Optical modelling

ZEMAX software (ANSYS, Inc., Canonsburg, PA) was used to combine lens geometry and GRIN to create the optical model of the bovine lens used in this study. The bovine lens was modelled as a doublet design with an anterior and posterior GRIN surface. As done previously in our human and mouse studies (Pan, et al., 2019, 2023), the boundary between these two surfaces was defined as the plane through the "optical centre" of the lens. The GRIN profiles were modelled using GRIN 3 in the ZEMAX glasses library (Pan et al., 2019). The respective anterior and posterior lens geometry and GRIN profiles were inputted into these two surfaces to create an optical model of the biological lens. Other parameters used to formulate the model included a pupil diameter of 2 mm, a polychromatic light source (wavelengths = 486, 587, and 656 nm), and field weighting of 0° = 100 %, 2.5° = 40 %, and 5° = 20 % (Vaghefi et al., 2015; Lim et al., 2016). After reconstructing the initial model, we optimised the model using contrast as a criterion to find the optimal focal length. The optical power of the lens, expressed in dioptres (D), and its effective focal length (in millimetres) were extracted from the model using the power field map function in ZEMAX.

## 3. Results

In our previous MRI-based studies, which utilised organ-cultured bovine lenses, we employed a small-bore 4.7T animal MRI scanner (Vaghefi, et al., 2011, 2015; Lim et al., 2016; Vaghefi and Donaldson, 2018). In this system, due to the small bore of the scanner, we could only accommodate two lenses, a control lens and a test lens, per experiment. In the present experiments, we have developed a new sample holder capable of accommodating multiple lenses and compatible with a clinical 3T scanner, allowing us to apply imaging protocols suitable for *in vivo* human lens imaging. We first describe the optimisation of this imaging approach before presenting its ability to measure steady-state water content and its changes in response to exposure to a hypotonic challenge designed to increase lens water content.

### 3.1. Development of a multi-lens MRI-compatible sample holder

To increase the number of experiments that can be conducted in a single MRI-based assay, we have developed an MRI-compatible sample holder that allows for the organ-culture of up to 12 lenses (Fig. 1A). This holder fits inside a 16-channel hand/wrist coil (Fig. 1A), which is then placed inside a clinical 3T MRI scanner. The initial holder prototypes, fabricated from polylactic acid (PLA) filaments (Fig. 1B, *top panels*), produced MRI susceptibility artefacts caused by local magnetic field distortions at material/tissue interfaces (Schenck, 1996). These artefacts were particularly pronounced at longer TE values (Czervionke et al., 1988). In our system, we found these artefacts arise from two sources: the air/sample interfaces around the edge of the holder and from the material used to construct the holder. Through trial and error, we found that susceptibility artefacts in our scans were effectively eliminated by incorporating a water channel into the holder to remove air/sample interfaces near the lenses, and by constructing the holder from PMMA, a low-density, MRI-compatible material (Fig. 1B, *bottom panels)*.

Using this holder design, it is possible to scan lenses in an equatorial orientation and collect images from all 12 lenses in a single scan (Fig. 1A, *bottom panel*). However, to collect images of the anterior and posterior surface curvatures, lens needed to be scanned in an axial orientation through the optical axis, which necessitated scanning each column of the holder in turn (Fig. 1C). However, this approach was initially not totally effective because the lenses were not consistently aligned within each column of the holder (Fig. 1C, *top panel*). To overcome this problem, the holder was redesigned with a depression that mimics the posterior curvature of the lens to ensure consistent alignment of the lenses within each column of the holder (Fig. 1C, *bottom panel*). This design improvement also helped prevent the floating of individual lenses, thereby also enhancing the consistency of scans oriented through the equatorial region of the lens (Fig. 1C). This optimised chamber design was then used to test the ability of the MPM protocol to simultaneously extract measurements of the steady-state total, free, and bound water in multiple lenses that are organ-cultured in isotonic AAH.

### 3.2. Measurement of lens water content using MPM protocols

Since previous studies have shown that a gradient of free water content exists in the lens, which contributes to the high refractive index observed in the central lens nucleus, we first investigated whether our multi-parameter protocols could spatially map the relative relationships between the different states of water that contribute to the GRIN (Pierscionek and Regini, 2012; Donaldson et al., 2023). These contributions are visualised as 2D colour maps of PD, MT ratio, T1, and T2* taken through the equatorial plane (Fig. 2A) and axial plane (Fig. 2B) of a representative bovine lens incubated in AAH. In these colour maps, blue represents the lowest values and red represents the highest values of the measured parameter, with the colour gradient illustrating spatial variations across the outer cortex (OC), inner cortex (IC), and nucleus (N).

To obtain an average of parameters from multiple lenses, line profiles were obtained through the centre of equatorial sections from multiple lenses (N = 12), averaged, and the resulting PD, T1, MT ratio, and T2* profiles plotted against normalised distance (*r/a*) from the lens centre (Fig. 2C). PD (total water) was lowest in the nucleus and increased steadily towards the periphery. A similar trend was observed for T1 (free water), with a plateau across the inner regions (N to IC) followed by a sharp rise in the OC region. By contrast, the MT ratio (bound water) showed the opposite pattern, with the nucleus exhibiting the highest signal, which decreased towards the periphery. T2* (water-to-protein ratio) followed the T1 profile, but with a more pronounced central plateau than observed for the other parameters.

To test the reproducibility of the high-throughput MPM protocols, two separate experiments were conducted with a separate batch of lenses (N = 12) each on different days. The lenses were pre-incubated for 2 h, then kept under the same conditions, and subsequently scanned using the customised sample and MPM protocols. Table 2 reports the summary statistics of two experiments. An independent-sample test reveals no statistically significant differences between the two experiments, indicating consistent outcomes across independent batches of lenses. The coefficient of variation (CoV) is generally lower (*<*10 %) for multiple parameters. T1 & PD show low coefficients of variance (CoV), which indicates excellent inter-sample precision. MT measurements show the moderate CoV across all lens regions. T2* shows the highest CoV (~12–31 %), which is probably because T2* is more sensitive to the field inhomogeneity.

In our previous studies, we showed that T1 and T2 are directly related to each other and inversely related to the GRIN. To illustrate the relative contributions of different water states to each other in the different regions of the lens, correlation plots were generated (Fig. 3). These plots showed an inverse correlation between free water (T1) and bound water (MT ratio) across lens regions (Fig. 3A), with bound water being most abundant in the nucleus, where protein concentration and refractive index are highest. In this study, T2* was used as a surrogate for the previously used T2 parameter, which reflects the water-to-bound protein ratio. Accordingly, T2* was plotted as 1/T2*, given its inverse relationship with the refractive index (Jones et al., 2005). Free water (T1) was lowest in the lens nucleus (Fig. 3B), where the refractive index (1/T2*) was highest. Moreover, 1/T2* was positively correlated with bound water (MT ratio), with the highest values observed in the nucleus (Fig. 3C). Taken together, these findings suggest that the MPM protocol effectively determines the regional differences in the state of water in the bovine lens and suggest that 1/T2* and MT are related to the GRIN.

### 3.3. Calculation of lens GRIN and lens power using the MPM protocol

**Fig. 4.**
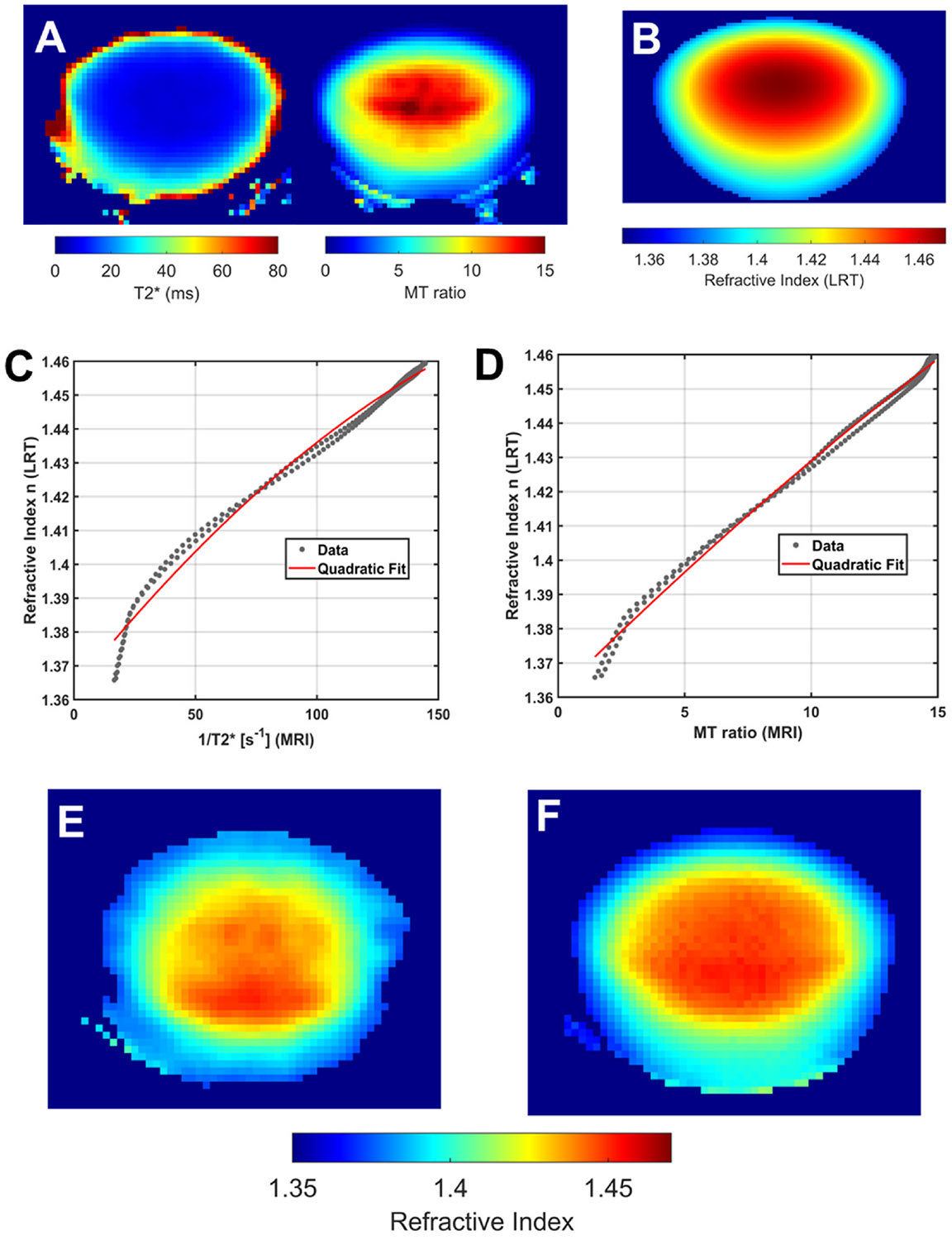
Calculation of the gradient of refractive index (GRIN) from MPM parameters. (**A & B**) Representative T2* (left) and MT (right) maps were correlated with the GRIN map (**B**) reconstructed using laser ray tracing (LRT) to produce the plots shown in panels **C** & **D**. (**C & D**) Plots of 1/T2* (**C**) and MT (**D**) against the refractive index obtained from LRT were fitted with quadratic fits to yield Equations (3) and (4) that converted T2* and MT values, respectively, to refractive index *(n*) values. (**E & F**) The resultant calibrations were used to convert T2* (**E**) and MT (**F**) maps into GRIN maps. The colour maps show that *n* is the lowest at the lens outer cortex (blue) and the highest at the lens nucleus (red).

To further expand the utility of our MPM protocol, we established a method to convert MRI-derived T2* and MT ratios (Fig. 4A) into refractive index maps, which could then be used in optical models to calculate lens power. This conversion is based on the concept that refractive index (*n*) is inversely correlated with the water-bound-protein ratio (T2), and this approach has been successfully used to establish T2-*n* calibration curves for bovine, mouse, and human lenses. In this study, we utilised a laser ray tracing (LRT) system developed in our laboratory to generate a map of the refractive index from organ-cultured bovine lenses (Fig. 4B). To generate the calibration curve, line profiles of T2* and *n* values were extracted from the same equatorial plane and plotted against their corresponding *r/a* values (Fig. 4C). The resultant T2*-*n* calibration is described by equation (3):

(3) $$n=-0.0167\times 10^{-4}\times {\left(\frac{1}{T2^{*}}\right)}^{2}+0.895\times 10^{-3}\times \left(\frac{1}{T2^{*}}\right)+1.3631$$

Where *T*2* is in seconds. The goodness of fit, R^2^, was 0.980.

Since 1/T2* is also related to MT (Fig. 3C), we also used the GRIN profile generated by LRT to produce an MT-*n* calibration curve (Fig. 4D). Several mathematical models were evaluated to fit the relationship between MT and *n*, and a second-order polynomial (equation (4)) provided the most accurate fit (R^2^ = 0.995) and was therefore used as the calibration equation to convert the MT ratio to *n*.


(4) $$n=-0.001\;MT^{2}+0.0073\times MT+1.3613$$

The resultant calibration equation (3) and (4) were then used to synthesise a GRIN map from a measured T2* (Fig. 4E) and MT ratio (Fig. 4F), respectively. The map regenerated from the T2* data sets appeared to have a higher variability than maps generated using the MT ratio. Because the MT-*n* calibration exhibited a higher R^2^ value and produced lower variation, we only used the MT-*n* calibration for the subsequent ZEMAX modelling.

By combining the MT-derived GRIN maps with the measurements of the lens in the optical modelling platform ZEMAX the optical properties of the bovine lens could be calculated (Vaghefi et al., 2015; Lim et al., 2016). Lastly, the lens power measured from a group of six lenses, estimated using MPM protocols, produces a mean power of 33.75 ± 1.43D and an effective focal length of 29.68 ± 1.29 mm. Their values agreed well with our previous LRT measurement of AAH-incubated lens, which had a mean power of 34.57 ± 2.8D and an effective focal length of 29.12 ± 2.28 mm.

### 3.4. Effect of swelling the lens on water content, GRIN and power

**Fig. 5.**
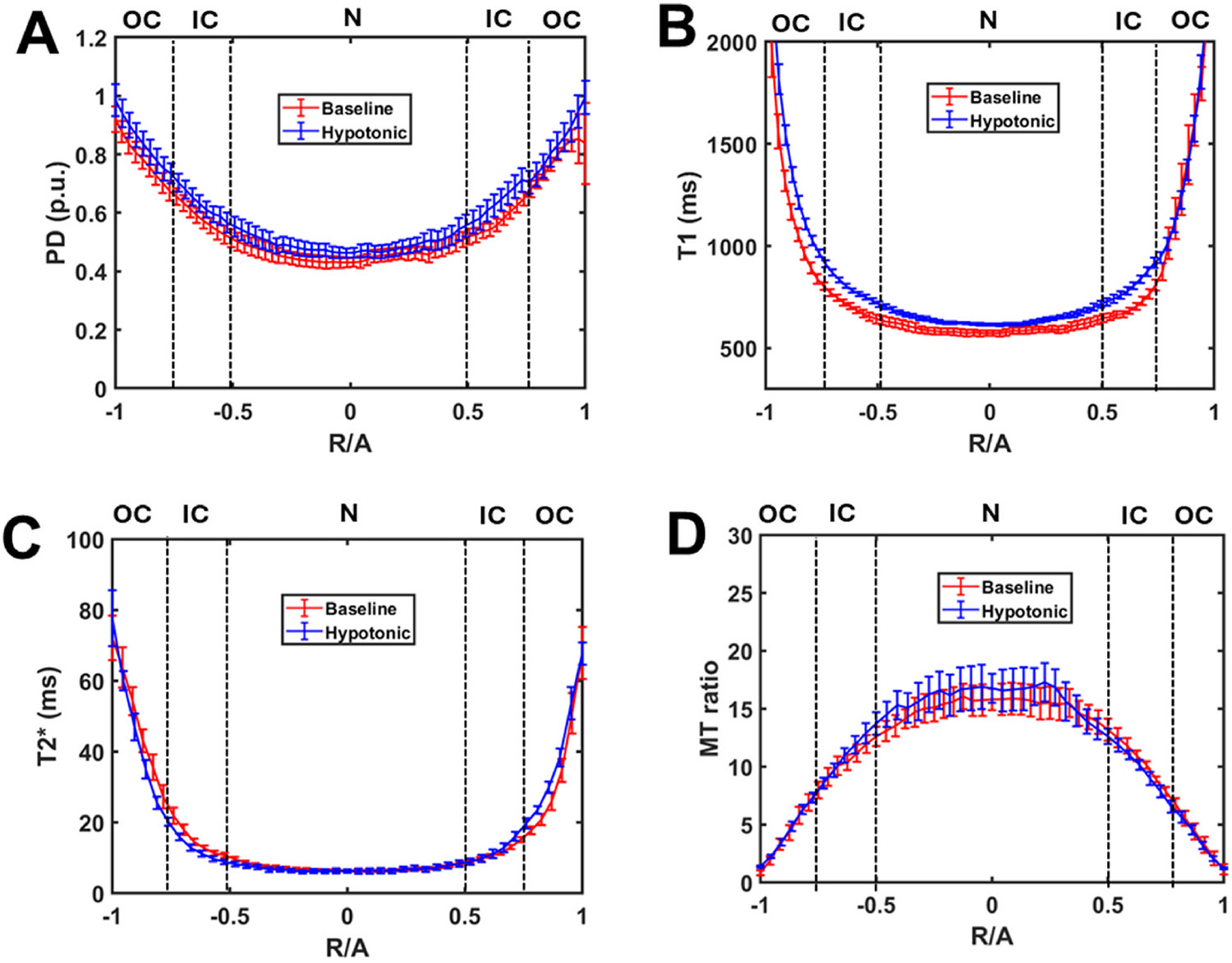
Effect of hypotonic challenge on lens water content measured using MPM protocols. Trend profiles extracted from PD (**A**), T1 (**B**), T2* (**C**), and MT ratio (**D**) maps from six lenses incubated in isotonic AAH (*red*) and then again after incubation for 2 h in hypotonic AAH solution (*blue*). OC: outer cortex; IC: inner cortex; N: nucleus.

To demonstrate the utility of our new system to detect changes in lens water content that affect the optical properties of the lens, we applied our MPM protocols to multiple lens exposed to a hypotonic challenge designed to swell the lens and increase lens water content. Lenses were initially scanned in AAH to establish baseline values for T1, PD, T2* and MT, before incubating the same lenses in hypotonic AAH at 37 °C for 2 h. Exposure to a hypotonic challenge resulted in the expected swelling of the lens, which was quantified as a 10 % decrease in the anterior radius of curvature, a 6.7 % decrease in the posterior radius of curvature, and a 5.3 % increase in lens thickness (Table 3). Fig. 5 illustrates the trend analysis comparing all parameters at baseline and after 2 h of hypotonic incubation. Compared with the baseline measurement, the total water content (PD) increased modestly in all regions, which showed that the change in lens geometry was driven by the expected gain in lens water (Fig. 5A). Furthermore, this increase in total water was attributed to a rise in the free water content, as indicated by the significant increase in T1 in all lens regions (Fig. 5B). In contrast, the MT ratio (Fig. 5C) and T2* (Fig. 5D) profiles showed no significant changes in any lens region before or after hypotonic exposure (Table 3), indicating that the bound water fraction remained relatively stable under hypotonic stress.

**Fig. 6.**
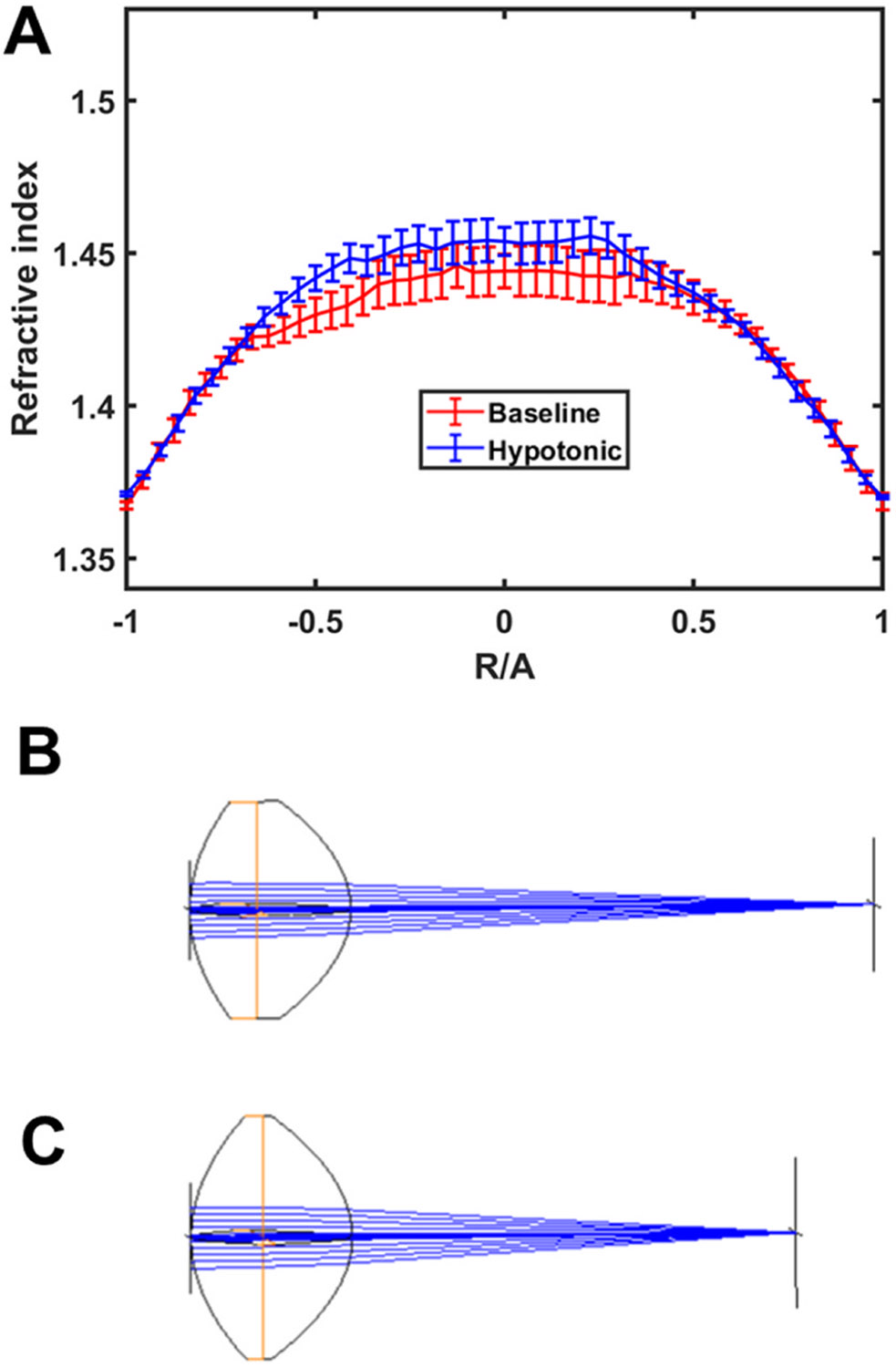
Effect of hypotonic challenge on the GRIN and power of the bovine lens. (**A**) Average refractive index profiles calculated from bovine lenses (n = 6) incubated in AAH (*red*) and then after a 2-h exposure to hypotonic AAH (*blue*). (**B & C**) Lens geometry and GRIN maps were extracted from lenses incubated in isotonic (**B**) and hypotonic (**C**) AAH and inputted into optical modelling software to construct optical models of the bovine lens under the two different conditions. Lenses exposed to hypotonic AAH exhibited a shorter focal length, indicating an increase in optical power.

Taken together, these results indicate that exposure to hypotonic conditions causes the lens to take up water and swell without altering the fraction of bound water. Consistent with this, hypotonic challenge produced minimal change in the GRIN profile derived from the MT-*n* relationship (Fig. 6A). However, the swelling-induced change in lens geometry increased optical power and shortened focal length relative to lenses incubated in isotonic AAH (Fig. 6B and C). These proof-of-principle experiments demonstrate that the multi-lens MRI platform can sensitively detect regional changes in lens water state and their corresponding effects on overall optical performance.

## 4. Discussion

Through the application of an MRI-based optical modelling approach, developed initially using *ex vivo* bovine lenses (Vaghefi et al., 2015), and then subsequently in *in vivo* studies that utilised transgenic mice (Pan et al., 2024) and human subjects (Lie, et al., 2021, 2024), we have shown that water content can be used as a biomarker of the age-related changes to lens optics that precede presbyopia and cataract development. This study revisits the *ex vivo* bovine lens as a preclinical model to assess its suitability for screening agents that, by modulating lens water content, may serve as therapeutic candidates to delay the onset of presbyopia and cataract. In this current study, we took the first steps towards this goal by developing and validating an MRI-based optical modelling approach that can accommodate the multiple bovine lenses needed for efficient screening for reagents that regulate lens water transport.

The first step in this process was to design a new MRI-compatible sample holder that could hold multiple lenses and which did not suffer from susceptibility artefacts (Fig. 1). This was achieved by incorporating a water channel into the holder to remove the air/sample interfaces close to the lenses and by constructing the chamber from PMMA, an MRI-compatible material widely used in various MRI applications (Wapler et al., 2014; Belzberg et al., 2019). Furthermore, to prevent lens movement during scanning and to facilitate image alignment when collecting axial views of the lens (Fig. 1C), the bottom of the holder was shaped to match the posterior pole of the lens. This chamber fitted into a wrist coil, which was then placed into a clinical 3T scanner, and the lenses were imaged using MPM protocols.

Previous MRI experiments had utilised a combination of different imaging protocols to obtain either T1 or T2 values from bovine lenses, and these protocols required lengthy acquisition times (Vaghefi et al., 2015; Lim et al., 2016). In contrast, the multi-parametric mapping (MPM) protocol developed in this study enabled simultaneous acquisition of proton density (PD), T1, T2* (as a surrogate for T2), and magnetisation transfer (MT) ratio across multiple lenses within a single scan protocol. This approach substantially reduced total scan time and improved inter-sample reproducibility (Table 2). In MPM, the MT-weighted sequence applies off-resonance energy to saturate the bound proton pool associated with macromolecules. This saturation transfers to the free water pool, reducing its signal. The resulting MT ratio therefore provides an indirect measure of the macromolecular or bound water fraction within the lens (Wolff and Balaban, 1989). With the inclusion of the MT ratio as an indicator of bound water, the MPM protocol provides a comprehensive picture of all the various states of water within the lens.

T1 has been considered as an indicator of the free water content of the lens in numerous studies (Vaghefi et al., 2015; Lie et al., 2021; Pan et al., 2023). In these studies, the extracted T1 profiles showed elevated values at the periphery and reduced values at the core of the lens, a pattern also observed using the MPM protocol (Fig. 2C). PD measures the total protons in a tissue, and is therefore used as a measure of both free water and water bound to proteins (Tofts, 2003; Lie et al., 2021). PD profiles have been measured in humans (Lie, et al., 2021, 2024) and rat lenses (Dobretsov et al., 2013) to determine their total water content. In the human lens, the normalised PD profile of the lens was constant across all regions and did not change with age (Lie et al., 2021). However, in the rat (Dobretsov et al., 2013) and now in the bovine lens (Fig. 2C), the PD pattern appears to follow the trend of free water, although with a shallower gradient than that observed in free water measurements. Consistent with this relationship between PD and T1, swelling the lens by exposure to hypotonic challenge increased both the total (Fig. 5A) and free (Fig. 5B) water content.

T2 has been used extensively to measure the water-to-protein ratio in various species of lens (Vaghefi et al., 2015; Pan, et al., 2019, 2023; Muir et al., 2020). In the MPM protocol, T2*, which is proportional to the transverse relaxation time T2 (Chavhan et al., 2009), is the measured parameter. In the bovine lens, T2* (Fig. 2C) and T2 (Lim et al., 2016) produce equivalent profiles of similar shape, both of which detect a central plateau in the water to protein ratio. This study is the first time MT has been measured in the lens; however, the parameter has been measured in the brain, where it is used as an indicator of demyelination, which is associated with a loss of bound water from myelin (Callaghan et al., 2014; Ziegler et al., 2018). The MT profile observed in bovine lenses was lowest in the periphery and highest in the lens nucleus (Fig. 2C) and exhibited an inverse relationship to T1 (Fig. 3A), which is consistent with the patterns of free and bound water found by others in the lens (Fagerholm et al., 1981; Tabandeh et al., 1994). Hence, the introduction of the MT ratio as a tool to monitor changes in bound water content is an important development that could, in the future, be used to study how bound and free water are altered with ageing (Heys et al., 2008), thereby helping to elucidate the pathophysiological mechanisms underlying age-related cataract formation.

Previous studies had converted T2 profiles into the GRIN measurements by using a set of calibrations (Jones et al., 2005; Vaghefi et al., 2015; Pan et al., 2019; Muir et al., 2020). These calibrations were then applied to generate GRIN plots that can be used for optical modelling of the lens (Pan et al., 2019; Lie et al., 2025). In this present study, we have trialled the use of the parameter T2*, produced by the MPM protocol, as a surrogate for T2 in the calculation of the GRIN. In addition, since 1/T2* was strongly related to MT (Fig. 3C), we investigated whether both parameters could be used to generate GRIN maps by establishing calibration curves for 1/T2* and MT using GRIN maps obtained from LRT experiments (Fig. 4). Consistent with the previously established 1/T2–*n* calibration (Jones et al., 2005; Muir et al., 2020), a non-linear relationship was observed between T2* and *n* (Fig. 4C), which was well characterised by a second-order polynomial equation (Equation (3)). A similar relationship was observed between MT ratio and *n* (Fig. 4D), which was also best fitted by a second-order polynomial equation (equation (4)) and showed a higher correlation coefficient than the 1/T2*-*n* calibration. The stronger correlation of the MT-*n* calibration curve is consistent with the notion that MT more directly reflects the bound water pool within the lens, which forms the basis of the GRIN distribution. Furthermore, compared with T2*, MT is less affected by field inhomogeneity and magnetic susceptibility artefacts. Hence, for these biological and technical reasons, we preferred MT-derived maps of the GRIN for use in optical modelling to calculate lens power.

By combining the GRIN maps with measurement of lens geometry into our established ZEMAX-based optical modelling platform (Pan et al., 2019), we were then able to calculate lens power and effective focal length for lenses incubated in either AAH or Hypotonic AAH (Fig. 6). Using the MT-*n* calibration to calculate the GRIN, we showed that the mean power and effective focal length (Table 3) were consistent with our previous LRT study on bovine lenses incubated in isotonic AAH (Qiu et al., 2017). Hypotonic challenge, which caused the lens to gain water (Fig. 5A and B) and swell (Table 3), did not significantly change the GRIN profile (Fig. 6A), a result consistent with the lack of effect hypotonic challenge had on T2* (Fig. 5C) and MT (Fig. 5D). The change in lens geometry did however cause an increase in lens power and thus a reduction in focal length (Fig. 6B and C). Hence, the lens water and power changes induced by hypotonic challenge illustrate the capability of our multi-parametric imaging approach to capture how alterations in lens water contents influence optical properties.

In summary, we have improved our MRI-based optical modelling approach to allow the simultaneous monitoring of the effects of interventions that alter lens water content and refractive power across multiple bovine lenses maintained in organ culture. In future work, this platform will allow us to screen for novel pharmacological interventions which by modulating the cellular physiology of lens water transport act to protect the lens against the progression of presbyopia and cataract in humans.

## Figures and Tables

**Table 1:** Imaging parameters for MPM protocol.

Protocol	Parameters					Description
	TR (ms)	TE (ms)	FA (°)	Voxel Size (mm)	Bandwidth (Hz/Pixels)	
**B1**^+^ **map**	30000	2.11	8	1.125 × 1.125 × 2	600	turbo-FLASH with saturation preparation
**T1/PD dual flip angle**	15	2.49	[4, 23]	0.21 × 0.21 × 2	279	VIBE
**PD weighted (PDW)**	23	[2.46, 4.92, 7.38, 9.84]	4	0.4 × 0.4 × 2	475	multi-echo FLASH
**T1 weighted (T1W)**	23	[2.46, 4.92, 7.38, 9.84]	23	0.4 × 0.4 × 2	475	multi-echo FLASH
**MT weighted (MTW)**	37	[2.46, 4.92, 7.38, 9.84]	9	0.4 × 0.4 × 2	475	multi-echo FLASH with additional MT preparation

**Table 2:** Summary statistics of the inter-sample reproducibility test.

Lens Region	Parameters	Experiment 01		Experiment 02		
		Mean (std)	CoV (%)	Mean (std)	CoV (%)	p-value
**OC**	PD	0.71 (0.02)	2.78	0.70 (0.014)	2.05	0.23
	T1 (ms)	919.7 (60.0)	6.53	917.7 (41.1)	4.48	0.93
	T2* (ms)	19.52 (4.36)	22.35	23.03 (7.21)	31.33	0.17
	MT	7.23 (0.68)	9.75	6.98 (0.48)	6.82	0.30
**IC**	PD	0.55 (0.01)	2.52	0.544 (0.01)	2.13	0.09
	T1 (ms)	746.7 (54.5)	7.30	763.2 (23.2)	3.04	0.35
	T2* (ms)	11.16 (2.45)	21.91	11.34 (2.95)	25.99	0.87
	MT	10.79 (1.53)	14.18	9.993 (0.61)	6.07	0.13
**N**	PD	0.47 (0.01)	1.66	0.46 (0.01)	1.32	0.09
	T1 (ms)	675.6 (33.1)	4.89	679.5 (21.0)	3.09	0.74
	T2* (ms)	8.39 (0.99)	11.83	8.32 (0.95)	11.41	0.85
	MT	12.43 (0.87)	6.97	11.96(0.50)	4.17	0.12

Note: OC: outer cortex; IC: inner cortex; N: nucleus; PD: proton density; MT: magnetisation transfer.

**Table 3:** Summary statistics of the hypotonic-treated lens.

Lens Region	Parameters		Baseline	Hypotonic 2 h			
		Mean (std)	CoV (%)	Mean (std)	CoV (%)	Δ Mean^a^	p-value
**OC**	PD	0.68 (0.03)	5.00	0.74 (0.04)	4.80	0.07	0.021†
	T1 (ms)	838.67 (52.38)	2.80	912.57 (16.50)	1.80	73.90	0.001†
	T2* (ms)	16.91 (4.91)	23.30	15.93 (2.12)	13.30	−5.98	0.17
	MT ratio	7.99 (1.61)	16.20	8.58 (1.03)	16.21	0.59	0.115
**IC**	PD	0.51 (0.03)	6.00	0.57 (0.04)	6.10	0.06	0.027†
	T1 (ms)	644.07 (28.31)	7.50	731.08 (11.65)	4.40	87.01	*<*0.001†
	T2* (ms)	7.70 (1.31)	13.50	7.74 (0.62)	117.90	−1.96	0.87
	MT ratio	13.96 (1.53)	14.18	14.52 (0.61)	6.07	2.56	0.13
**N**	PD	0.43 (0.02)	1.66	0.47 (0.02)	1.32	0.04	0.01†
	T1 (ms)	573.46 (28.31)	4.90	615.20 (11.65)	1.90	41.75	0.001†
	T2* (ms)	6.40 (0.40)	6.30	6.35 (1.69)	26.60	−0.05	0.89
	MT ratio	15.75 (0.87)	9.30	16.66 (0.50)	4.17	0.91	0.37
**Geometry**	Ra (mm)	10.13 (0.40)	3.90	9.10 (1.07)	11.94	−1.00	0.02†
	Rp (mm)	6.34 (0.57)	12.12	5.92 (0.72)	12.16	−0.42	0.047†
	LT (mm)	12.80 (1.06)	8.26	13.51 (0.78)	5.80	1.51	0.01†
**Optics**	EFL (mm)	29.68 (1.29)	4.35	28.21 (2.33)	8.25	−1.47	0.04†
	Power (D)	33.75 (1.43)	6.43	36.73 (3.92)	10.67	2.98	0.04†

α.Δ Mean is the mean difference of using a hypotonic-treated lens minus the baseline.

† Indicating statistically significant (p *<* 0.05).

Note: OC: outer cortex; IC: inner cortex; N: nucleus; PD: proton density; MT: magnetisation transfer.

## Data Availability

No data was used for the research described in the article.
